# Supplementary material for: Effects of Genetic Mutation Sites in ADR Genes on Modern Chickens Produced and Domesticated by Artificial Selection
Source: Biology (Basel). 2023 Jan 20;12(2):169. doi: 10.3390/biology12020169 (PMC9952598; doi:10.3390/biology12020169)
Supplement: Supplementary file 1 [file biology-12-00169-s001.zip › TableS1_S12_Ver.3.pdf]

Table S1: List of primers for the nine ADR gene sequences.

Gene Name	Primer No.	Forward	Primer No.	Reverse
ADR A1A Ex1	F437	TGGTGGCTATTAGCTGTGCTAGA	R1767	TTCCCTTTGAACCAGACCTGCTTG
ADR A1A Ex2	F3	AGGCTTCGAGTCATAAATTTCCCA	R758	TATTTGTTTCATCCCAACCCCTTCA
ADR A1B Ex1	F35	AACTGAACTCAACCATCTGCGTGTT	R1209	TTCACTTCCCACCCTTTCATCTGGT
ADR A1B Ex2	F57	AATTGAAAGCAGCACATGTTAGGGA	R2188	TGGCTTCCTTCTAGTGGCATACTG
ADR A1D Ex1	F9	GCTGGGAGATCACTCCATCCCTACT	R1291	TCACTGCTGGTTTCTTGACCGATCT
ADR A1D Ex2	F20	ACACTAAGGAAGCTCCCACACCT	R389	GGTAGCCTTTGGAAGGGCTGAATCC
ADR A1D Ex3	F40	TGGCTGTCCACCAGCCTTGCTACAC	R2307	AATCTGCCTGACAGACCCACAGA
ADR A2A_1	F102	TAGGAGCGCGGAGGTCCGCGGAGA	R1636	TCACAAGCTGAAGGAAACCATCCCAT
ADR A2A_2	F502	ACATCCTGGTGGCCACGCTGGTCAT	R1215	TGGGCAAAGTGTCCCCAGGCTTAAT
ADR A2B_1	F238	AGTGCCCCAATACCACGGTGGAAGCT	R904	GCAGGATGAGGACGGCCACCAGGAT
ADR A2B_2	F880	ATCCTGGTGGCCGTCCTCATCCTGC	R1793	CGCTTCTGGAAGTACCTTGACTTCA
ADR A2C	F9	TCCGCGGGGCTCTGTAGGACGGCG	R1463	GCACAGAGCCTTTCCCTTCCATTTTAA
ADR B1_1	F888	AACGCCGTGCGCTGCACACCGAGGAGG	R2618	TGGGCTTCCTGTTTGCCTTAGGTTC
ADR B1_2	Ex2_Ex3_F	GCAATAAATGGGCCGTGTTTATATTAA	Ex2_Ex3_R	TAACACGGATCTATTTACAGTACCTTC
ADR B1_3	F_END1	TGAGTTGTTGTGCTTATCTCTTTGGCCA	R_END2	AACCTCCATTTCACTTTTAATTTGCGTG
ADR B1_4	0609_F	GGGAGTTAATGAGCCTTCTGCAGCTTGTTC	0609_R	GTGTGTGGACGTGTGCAAGTAGCATCTTCA
ADR B1_5	1270_F	ATGAGCCTTCTGCAGCTTGTTCG	1270_R	TGCGTGTGTGGACATGTGCAAGTAG
ADR B2	F1	ACCTGCTGTCCCCTATGCGCGGA	R2	GCCATTGGGGTTTAGGTGTGCT
ADR B3 Ex1_1	F757	TGCAGCCTCGACGGCGGCGGCCGC	R1859	AGCACAGCAGCTTGCGGAAAGCGCT
ADR B3 Ex1_2	F1478	TCCACCATCTCCTTCTACGTGCC	R2216	GCCCGCTCACCAGCTCTGCATTCA
ADR B3 Ex2	F2443	TACTGGGCCACCAGGAGTGTGTTC	R2704	CTGCTGCAGGGACAGGGGTGGCCC

Table S2. Accession number of nine adrenergic receptors of wild birds.

ADRB1：
XM 015867370.2 PREDICTED: Coturnix japonica adrenoceptor beta 1 (ADRB1) mRNA
NM 001303175.1 Meleagris gallopavo adrenoceptor beta 1 (ADRB1) mRNA
XM 021400335.1 PREDICTED: Numida meleagris adrenoceptor beta 1 (ADRB1) mRNA
XM 031597451.1 PREDICTED: Phasianus colchicus adrenoceptor beta 1 (ADRB1) mRNA
LC720796.1 Gallus gallus gallus 222 ADRB1 beta-1 adrenergic receptor, complete cds
ADRA1B:
XM 015876066.2:479-2002 PREDICTED: Coturnix japonica adrenoceptor alpha 1B (ADRA1B) mRNA
XM 003210292.4:490-2013 PREDICTED: Meleagris gallopavo adrenoceptor alpha 1B (ADRA1B) mRNA
XM 021410804.1:545-2068 PREDICTED: Numida meleagris adrenoceptor alpha 1B (ADRA1B) mRNA
XM 031598056.1:475-1998 PREDICTED: Phasianus colchicus adrenoceptor alpha 1B (ADRA1B) mRNA
LC720797.1 Gallus gallus gallus 222 ADRA1b alpha-1B adrenergic receptor, complete cds
ADRB3:
XM 015883180.2:19-1405 PREDICTED: Coturnix japonica adrenoceptor beta 3 (ADRB3) mRNA
NM 001303183.1:112-1337 Meleagris gallopavo adrenoceptor beta 3 (ADRB3) mRNA
XM 021374766.1:1-1367 PREDICTED: Numida meleagris adrenoceptor beta 3 (ADRB3) mRNA
XM 031602247.1:136-1363 PREDICTED: Phasianus colchicus adrenoceptor beta 3 (ADRB3) mRNA
LC720801.1 Gallus gallus gallus 222 ADRB3 beta-3 adrenergic receptor, complete cds
ADRB2:
XM 015876329.1 PREDICTED: Coturnix japonica adrenoceptor beta 2 (ADRB2) mRNA
XM 010719047.2:1-1185 PREDICTED: Meleagris gallopavo adrenoceptor beta 2 (ADRB2) partial mRNA
XM 021410489.1 PREDICTED: Numida meleagris adrenoceptor beta 2 (ADRB2) mRNA
XM 031598089.1:217-1425 PREDICTED: Phasianus colchicus adrenoceptor beta 2 (ADRB2) mRNA
LC720802.1 Gallus gallus gallus 222 ADRB2 beta-2 adrenergic receptor, complete cds
ADRA2B:
XM 015876372.2:198-1692 PREDICTED: Coturnix japonica alpha-2C adrenergic receptor-like (LOC107320469) mRNA
XM 010718981.3:2-1484 PREDICTED: Meleagris gallopavo alpha-2Da adrenergic receptor-like (LOC100550610) mRNA
XM 021410469.1:1-1419 PREDICTED: Numida meleagris alpha-2Db adrenergic receptor-like (LOC110405283) mRNA
XM 031597911.1:1-1408 PREDICTED: Phasianus colchicus alpha-2Da adrenergic receptor-like (LOC116231902) mRNA
LC720804.1 Gallus gallus gallus 222 ADRA2b alpha-2B adrenergic receptor, complete cds
ADRA1A:
XM 032448889.1 PREDICTED: Coturnix japonica adrenoceptor alpha 1A (ADRA1A) transcript variant X3 mRNA
XM 010723203.2 PREDICTED: Meleagris gallopavo adrenoceptor alpha 1A (ADRA1A) mRNA
XM 021375050.1 PREDICTED: Numida meleagris adrenoceptor alpha 1A (ADRA1A) mRNA
XM 031596973.1 PREDICTED: Phasianus colchicus adrenoceptor alpha 1A (ADRA1A) mRNA
LC720799.1 Gallus gallus gallus 222 ADRa1A alpha-1A adrenergic receptor, complete cds
ADRA1B:
XM 015876066.2:479-2002 PREDICTED: Coturnix japonica adrenoceptor alpha 1B (ADRA1B) mRNA
XM 003210292.4:490-2013 PREDICTED: Meleagris gallopavo adrenoceptor alpha 1B (ADRA1B) mRNA
XM 021410804.1:545-2068 PREDICTED: Numida meleagris adrenoceptor alpha 1B (ADRA1B) mRNA
XM 031598056.1:475-1998 PREDICTED: Phasianus colchicus adrenoceptor alpha 1B (ADRA1B) mRNA
LC720797.1 Gallus gallus gallus 222 ADRA1b alpha-1B adrenergic receptor, complete cd
ADRA1D:
XM 015863039.2:391-2000 PREDICTED: Coturnix japonica adrenoceptor alpha 1D (ADRA1D) transcript variant X1 mRNA
XR 793488.3:385-1993 PREDICTED: Meleagris gallopavo adrenoceptor alpha 1D (ADRA1D) transcript variant X2 misc RNA
XM 021396914.1:361-1969 PREDICTED: Numida meleagris adrenoceptor alpha 1D (ADRA1D) mRNA
XM 031588716.1:159-1767 PREDICTED: Phasianus colchicus adrenoceptor alpha 1D (ADRA1D) mRNA
LC720798.1 Gallus gallus gallus 222 ADRA1D alpha-1D adrenergic receptor, complete cds
ADRA2A:
XM 032445492.1 PREDICTED: Coturnix japonica adrenoceptor alpha 2A (ADRA2A) mRNA
XM 010714708.3 PREDICTED: Meleagris gallopavo adrenoceptor alpha 2A (ADRA2A) mRNA
XM 021399617.1 PREDICTED: Numida meleagris adrenoceptor alpha 2A (ADRA2A) mRNA
XM 031597481.1 PREDICTED: Phasianus colchicus adrenoceptor alpha 2A (ADRA2A) mRNA
LC720800.1 Gallus gallus gallus 222 ADRA2a alpha-2A adrenergic receptor, complete cds

Table S3. The DNA and amino acid mutation sits of ADR genes from breeds.

	GeneName	Shaver Brown									Shamo								
		N5	N5	N5	N6	N6	N6	N7	N7	N7	S6	S6	S6	S7	S7	S7	S9	S9	S9
		DNA	Amino Acid	TMHMM	DNA	Amino Acid	TMHMM	DNA	Amino Acid	TMHMM	DNA	Amino Acid	TMHMM	DNA	Amino Acid	TMHMM	DNA	Amino Acid	TMHMM
1	ADR α1A exon1	—	—		—	—		—	—		—	—		—	—		—	—	
2	ADR α1A exon2	A1093R	S365G	inside	A1093R	S365G	inside	A1093R	S365G	inside	—	—		—	—		—	—	
3	ADR α1B exon1	—	—		G773R	R258Q	inside	—	—		—	—		—	—		—	—	
4	ADR α1B exon2	—	—		—	—		G1480A	V494M	inside	—	—		—	—		—	—	
5	ADR α1D exon1	—	—		—	—		—	—		—	—		T173K	L58W	TMhelix	—	—	
6	ADR α1D exon2	—	—		—	—		—	—		C1319M	T440N	inside	C1319M	T440N	inside	C1319M	T440N (C1319M)	inside
7	ADR α2A	—	—		G172R	V58I	outside	G172R	V58I	outside	G172A	V58I	outside	G172A	V58I	outside	G172R	V58I	outside
8		—	—		C819M	D273E	inside	—	—		C819M	D273E	inside	C819M	D273E	inside	—	—	
9	ADR α2A	—	—		—	—		—	—		—	—		—	—		G886R	V296I	inside
10	ADR α2B	—	—		—	—		—	—		G413R	R138Q	inside	—	—		—	—	
11		—	—		—	—		—	—		G629R	R210H	inside	—	—		—	—	
12	ADR α2B	—	—		—	—		—	—		—	—		G874R	V292M	outside	G874R	V292M	outside
13	ADR α2C	—	—		—	—		—	—		—	—		—	—		—	—	
14	ADR β1	G1208A	R403Q	inside	—	—		—	—		G1208A	R403Q	inside	G1208A	R403Q	inside	G1208A	R403Q	inside
15	ADR β1	—	—		—	—		—	—		G1328R	N443S	inside	G1328R	N443S	inside	G1328R	N443S	inside
17		—	—		—	—		—	—		G1334R	S445N	inside	G1334R	S445N	inside	G1334A	S445N	inside
18		—	—		—	—		—	—		C1396Y	R466C	inside	C1396Y	R466C	inside	C1396T	R466C	inside
16	ADR β1	—	—		G1330R	G444S	inside	—	—		—	—		—	—		—	—	
19	ADR β2	—	—		—	—		—	—		—	—		G43R	A15T	outside	G43R	A15T	outside
20	ADR β2	—	—		—	—		—	—		—	—		C131Y	T44I	Tmhelix	C131Y	T44I	Tmhelix
21		—	—		—	—		—	—		—	—		T132Y	T44I	Tmhelix	T132Y	T44I	Tmhelix
22		—	—		—	—		—	—		—	—		A695R	Q232R	inside	A695R	Q232R	inside
23		—	—		—	—		—	—		C830Y	T277M	Tmhelix	—	—		—	—	
24	ADR β3	C1024Y	R342C	inside	C1024Y	R342C	inside	C1024Y	R342C	inside	C1024Y	R342C	inside	C1024Y	R342C	TMhelix	C1024Y	R342C	inside
25		T1186Y	S396P	inside	T1186Y	S396P	inside	T1186Y	S396P	inside	T1186Y	S396P	inside	T1186Y	S396P	inside	T1186Y	S396P	inside
26		A1211W	Q404L	inside	A1211W	Q404L	inside	A1211W	Q404L	inside	A1211W	Q404L	inside	A1211W	Q404L	inside	A1211W	Q404L	inside
27		C1218M	P406S	inside	C1218M	P406S	inside	C1218M	P406S	inside	C1218M	P406S	inside	C1218M	P406S	inside	C1218M	P406S	inside

Orange indicates Shaver Brown-specific mutations, whereas blue indicates Shamo-specific mutations. Green indicates mutations common to both breeds. No color indicates external mutations.

Table S4. The protein subcellular localization prediction of nine ADR genes.

**alpha-1A adrenergic receptor [Gallus gallus] GenBank: BBK26311.1**				
id	site	distance	identity	comments
A1AA_HUMAN	plas	794.567	77.09%	[Uniprot] SWISS-PROT45:Integral membrane protein. GO:0005887; C:integral to plasma membrane; Evidence:TAS.
A1AA_ORYLA	plas	1640.35	57.89%	[Uniprot] SWISS-PROT45:Integral membrane protein.
O2W1_HUMAN	plas	2137.47	15.42%	[Uniprot] SWISS-PROT45:Integral membrane protein.
PF2R_HUMAN	plas	2294.05	14.78%	[Uniprot] SWISS-PROT45:Integral membrane protein. GO:0005887; C:integral to plasma membrane; Evidence:TAS.
EDG1_RAT	plas	2464.55	21.63%	[Uniprot] SWISS-PROT45:Integral membrane protein.
P2Y5_HUMAN	plas	2468.16	17.99%	[Uniprot] SWISS-PROT45:Integral membrane protein. GO:0016021; C:integral to membrane; Evidence:NAS.
TAR1_HUMAN	plas	2493.26	22.70%	[Uniprot] SWISS-PROT45:Integral membrane protein.
A1AA_RABIT	plas	2554.73	77.09%	[Uniprot] SWISS-PROT45:Integral membrane protein.
**alpha-1B adrenergic receptor [Gallus gallus] GenBank: BBK26315.1**				
id	site	distance	identity	comments
S1C1_MACFA	plas	8834.15	13.62%	[Uniprot] SWISS-PROT45:Integral membrane protein.
S1C1_HUMAN	plas	9028.57	16.15%	[Uniprot] SWISS-PROT45:Integral membrane protein.
ACM3_CAEEL	plas	9332.11	17.78%	[Uniprot] SWISS-PROT45:Integral membrane protein.
5HT_LYMST	plas	9385.45	24.50%	[Uniprot] SWISS-PROT45:Integral membrane protein.
ACM3_RAT	plas	9925.55	19.19%	[Uniprot] SWISS-PROT45:Integral membrane protein.
ACM3_MOUSE	plas	9934.19	19.19%	[Uniprot] SWISS-PROT45:Integral membrane protein.
D2DR_DROME	plas	10371.4	22.28%	[Uniprot] SWISS-PROT45:Integral membrane protein.
**alpha-1D adrenergic receptor [Gallus gallus] GenBank: BBK26322.1**				
id	site	distance	identity	comments
NY5R_CANFA	plas	960.224	17.61%	[Uniprot] SWISS-PROT45:Integral membrane protein.
ACM3_RAT	plas	970.06	20.37%	[Uniprot] SWISS-PROT45:Integral membrane protein.
NY5R_RAT	plas	1050.43	17.35%	[Uniprot] SWISS-PROT45:Integral membrane protein.
ACM3_MOUSE	plas	1072.92	20.54%	[Uniprot] SWISS-PROT45:Integral membrane protein.
NY5R_PIG	plas	1245.66	16.24%	[Uniprot] SWISS-PROT45:Integral membrane protein.
NY5R_HUMAN	plas	1294.31	16.96%	[Uniprot] SWISS-PROT45:Integral membrane protein. GO:0005887; C:integral to plasma membrane; Evidence:TAS.
NY5R_MOUSE	plas	1349.92	18.20%	[Uniprot] SWISS-PROT45:Integral membrane protein. GO:0016020; C:membrane; Evidence:IDA.
ACM3_GORGO	plas	1381.89	20.68%	[Uniprot] SWISS-PROT45:Integral membrane protein.
**alpha-2A adrenergic receptor [Gallus gallus] GenBank: BBK26327.2**				
id	site	distance	identity	comments
IRKF_CAVPO	plas	1142.91	14.61%	[Uniprot] SWISS-PROT45:Integral membrane protein.
CLT1_MOUSE	plas	1390.73	15.09%	[Uniprot] SWISS-PROT45:Integral membrane protein. GO:0005887; C:integral to plasma membrane; Evidence:IDA.
A1A4_MOUSE	plas	1640.41	12.21%	[Uniprot] SWISS-PROT45:Integral membrane protein. GO:0005890; C:sodium/potassium-exchanging ATPase complex; Evidence:ISS.
5H7_XENLA	plas	1656.39	22.93%	[Uniprot] SWISS-PROT45:Integral membrane protein.
A8B4_HUMAN	plas	1703.75	11.24%	[Uniprot] SWISS-PROT45:Integral membrane protein.
A1A4_RAT	plas	1850.33	11.67%	[Uniprot] SWISS-PROT45:Integral membrane protein. GO:0005890; C:sodium/potassium-exchanging ATPase complex; Evidence:ISS.
CCAM_MUSDO	plas	2256.16	8.18%	[Uniprot] SWISS-PROT45:Integral membrane protein.
IRK5_RAT	plas	2256.49	12.16%	[Uniprot] SWISS-PROT45:Integral membrane protein.
**alpha-2B adrenergic receptor [Gallus gallus] GenBank: BBK26332.1**				
id	site	distance	identity	comments
SSR5_RAT	plas	589.81	21.04%	[Uniprot] SWISS-PROT45:Integral membrane protein.
SSR5_MOUSE	plas	776.474	22.13%	[Uniprot] SWISS-PROT45:Integral membrane protein.
D3DR_CERAE	plas	871.177	32.19%	[Uniprot] SWISS-PROT45:Integral membrane protein.
D3DR_HUMAN	plas	920.785	32.43%	[Uniprot] SWISS-PROT45:Integral membrane protein. GO:0005887; C:integral to plasma membrane; Evidence:TAS.
GALS_HUMAN	plas	1234.47	22.56%	[Uniprot] SWISS-PROT45:Integral membrane protein. GO:0005886; C:plasma membrane; Evidence:TAS.
PE21_HUMAN	plas	1942.69	19.35%	[Uniprot] SWISS-PROT45:Integral membrane protein. GO:0005887; C:integral to plasma membrane; Evidence:TAS.
D3DR_MOUSE	plas	2128.75	28.26%	[Uniprot] SWISS-PROT45:Integral membrane protein.
D3DR_RAT	plas	2175.3	29.36%	[Uniprot] SWISS-PROT45:Integral membrane protein.
**alpha-2C adrenergic receptor [Gallus gallus] GenBank: BBK26341.1**				
id	site	distance	identity	comments
A1AA_BOVIN	plas	1973.97	24.74%	[Uniprot] SWISS-PROT45:Integral membrane protein.
A1AA_CAVPO	plas	2075.26	23.92%	[Uniprot] SWISS-PROT45:Integral membrane protein.
A1AA_RABIT	plas	2172.32	24.95%	[Uniprot] SWISS-PROT45:Integral membrane protein.
CCKR_XENLA	plas	2578.6	17.72%	[Uniprot] SWISS-PROT45:Integral membrane protein.
P2YC_MOUSE	plas	2821.8	14.77%	[Uniprot] SWISS-PROT45:Integral membrane protein. GO:0005887; C:integral to plasma membrane; IC.
PE24_RABIT	plas	2902.42	13.70%	[Uniprot] SWISS-PROT45:Integral membrane protein.
OPN4_MOUSE	plas	2953.32	19.00%	[Uniprot] SWISS-PROT45:Integral membrane protein.
PE24_RAT	plas	3064.75	12.91%	[Uniprot] SWISS-PROT45:Integral membrane protein.
**beta-1 adrenergic receptor [Gallus gallus] GenBank: BBK26345.2**				
id	site	distance	identity	comments
B1AR_MELGA	plas	107.217	94.20%	[Uniprot] SWISS-PROT45:Integral membrane protein.
A2AA_PIG	plas	786.115	24.69%	[Uniprot] SWISS-PROT45:Integral membrane protein.
A2AA_HUMAN	plas	942.004	24.11%	[Uniprot] SWISS-PROT45:Integral membrane protein. GO:0005887; C:integral to plasma membrane; Evidence:TAS.
A2AA_MOUSE	plas	986.248	24.43%	[Uniprot] SWISS-PROT45:Integral membrane protein.
A2AA_RAT	plas	998.854	24.84%	[Uniprot] SWISS-PROT45:Integral membrane protein.
CML2_HUMAN	plas	1121.49	15.72%	[Uniprot] SWISS-PROT45:Integral membrane protein. GO:0005887; C:integral to plasma membrane; Evidence:TAS.
A2AA_CAVPO	plas	1137.09	24.22%	[Uniprot] SWISS-PROT45:Integral membrane protein.
A2AA_BOVIN	plas	1240	24.48%	[Uniprot] SWISS-PROT45:Integral membrane protein.

beta-2 adrenergic receptor [Gallus gallus] GenBank: BBK26353.1

id	site	distance	identity	comments
B2AR_MACMU	plas	756.709	71.12%	[Uniprot] SWISS-PROT45:Integral membrane protein.
O4S1_HUMAN	plas	2013.5	16.62%	[Uniprot] SWISS-PROT45:Integral membrane protein.
O2Y1_HUMAN	plas	2331.67	17.38%	[Uniprot] SWISS-PROT45:Integral membrane protein.
NK2R_MOUSE	plas	2456.67	24.88%	[Uniprot] SWISS-PROT45:Integral membrane protein.
SPR1_MOUSE	plas	2584.04	19.55%	[Uniprot] SWISS-PROT45:Integral membrane protein.
B2AR_FELCA	plas	2614.15	72.73%	[Uniprot] SWISS-PROT45:Integral membrane protein.
B2AR_HUMAN	plas	2694.67	69.73%	[Uniprot] SWISS-PROT45:Integral membrane protein. GO:0005764; C:lysosome; Evidence:TAS.
SPR1_HUMAN	plas	2734.18	19.05%	[Uniprot] SWISS-PROT45:Integral membrane protein. GO:0005887; C:integral to plasma membrane; Evidence:TAS.

beta-3 adrenergic receptor [Gallus gallus] GenBank: BBK26359.1

id	site	distance	identity	comments
GALS_HUMAN	plas	1739.17	22.45%	[Uniprot] SWISS-PROT45:Integral membrane protein. GO:0005886; C:plasma membrane; Evidence:TAS.
MSHR_GORGO	plas	2003.14	21.65%	[Uniprot] SWISS-PROT45:Integral membrane protein.
MSHR_HUMAN	plas	2112.55	22.05%	[Uniprot] SWISS-PROT45:Integral membrane protein. GO:0005887; C:integral to plasma membrane; Evidence:TAS.
DBDR_HUMAN	plas	2204.08	30.27%	[Uniprot] SWISS-PROT45:Integral membrane protein. GO:0005887; C:integral to plasma membrane; Evidence:TAS.
MRGF_HUMAN	plas	2215.45	16.25%	[Uniprot] SWISS-PROT45:Integral membrane protein.
MSHR_ALOCA	plas	2230.97	22.67%	[Uniprot] SWISS-PROT45:Integral membrane protein.
MSHR_PANTR	plas	2314.4	21.60%	[Uniprot] SWISS-PROT45:Integral membrane protein.
O4D5_HUMAN	plas	2331.97	16.25%	[Uniprot] SWISS-PROT45:Integral membrane protein.

Table S5. Prediction of transmembrane helices in proteins of nine ADR genes.

ADRA1A alpha-1A adrenergic receptor [Gallus gallus] GenBank: BBK26311.1

BBK26311.1 Length: 467
BBK26311.1 Number of predicted TMHs: 7
BBK26311.1 Exp number of AAs in TMHs: 147.91708
BBK26311.1 Exp number, first 60 AAs: 23.36721
BBK26311.1 Total prob of N-in: 0.00031
BBK26311.1 POSSIBLE N-term signal sequence

BBK26311.1	TMHMM2.0	outside	1	27
BBK26311.1	TMHMM2.0	TMhelix	28	50
BBK26311.1	TMHMM2.0	inside	51	62
BBK26311.1	TMHMM2.0	TMhelix	63	85
BBK26311.1	TMHMM2.0	outside	86	99
BBK26311.1	TMHMM2.0	TMhelix	100	122
BBK26311.1	TMHMM2.0	inside	123	142
BBK26311.1	TMHMM2.0	TMhelix	143	165
BBK26311.1	TMHMM2.0	outside	166	184
BBK26311.1	TMHMM2.0	TMhelix	185	207
BBK26311.1	TMHMM2.0	inside	208	272
BBK26311.1	TMHMM2.0	TMhelix	273	295
BBK26311.1	TMHMM2.0	outside	296	309
BBK26311.1	TMHMM2.0	TMhelix	310	329
BBK26311.1	TMHMM2.0	inside	330	467

ADRA1B alpha-1B adrenergic receptor [Gallus gallus] BBK26315.1

BBK26315.1 Length: 507
BBK26315.1 Number of predicted TMHs: 7
BBK26315.1 Exp number of AAs in TMHs: 152.7034
BBK26315.1 Exp number, first 60 AAs: 9.82078
BBK26315.1 Total prob of N-in: 0.01195

BBK26315.1	TMHMM2.0	outside	1	51
BBK26315.1	TMHMM2.0	TMhelix	52	74
BBK26315.1	TMHMM2.0	inside	75	85
BBK26315.1	TMHMM2.0	TMhelix	86	108
BBK26315.1	TMHMM2.0	outside	109	122
BBK26315.1	TMHMM2.0	TMhelix	123	145
BBK26315.1	TMHMM2.0	inside	146	165
BBK26315.1	TMHMM2.0	TMhelix	166	188
BBK26315.1	TMHMM2.0	outside	189	207
BBK26315.1	TMHMM2.0	TMhelix	208	230
BBK26315.1	TMHMM2.0	inside	231	298
BBK26315.1	TMHMM2.0	TMhelix	299	321
BBK26315.1	TMHMM2.0	outside	322	335
BBK26315.1	TMHMM2.0	TMhelix	336	355
BBK26315.1	TMHMM2.0	inside	356	507

ADRA1D alpha-1D adrenergic receptor [Gallus gallus] GenBank: BBK26322.1

BBK26322.1 Length: 511
BBK26322.1 Number of predicted TMHs: 7
BBK26322.1 Exp number of AAs in TMHs: 154.20159
BBK26322.1 Exp number, first 60 AAs: 7.55847
BBK26322.1 Total prob of N-in: 0.00003

BBK26322.1	TMHMM2.0	outside	1	53
BBK26322.1	TMHMM2.0	TMhelix	54	76
BBK26322.1	TMHMM2.0	inside	77	87
BBK26322.1	TMHMM2.0	TMhelix	88	110
BBK26322.1	TMHMM2.0	outside	111	124
BBK26322.1	TMHMM2.0	TMhelix	125	147
BBK26322.1	TMHMM2.0	inside	148	167
BBK26322.1	TMHMM2.0	TMhelix	168	190
BBK26322.1	TMHMM2.0	outside	191	209
BBK26322.1	TMHMM2.0	TMhelix	210	232
BBK26322.1	TMHMM2.0	inside	233	300
BBK26322.1	TMHMM2.0	TMhelix	301	323
BBK26322.1	TMHMM2.0	outside	324	337
BBK26322.1	TMHMM2.0	TMhelix	338	357
BBK26322.1	TMHMM2.0	inside	358	511

ADRA2C alpha-2C adrenergic receptor [Gallus gallus] GenBank: BBK26341.1

BBK26341.1 Length: 446
BBK26341.1 Number of predicted TMHs: 7
BBK26341.1 Exp number of AAs in TMHs: 156.20257
BBK26341.1 Exp number, first 60 AAs: 15.51516
BBK26341.1 Total prob of N-in: 0.00292
BBK26341.1 POSSIBLE N-term signal sequence

BBK26341.1	TMHMM2.0	outside	1	45
BBK26341.1	TMHMM2.0	TMhelix	46	68
BBK26341.1	TMHMM2.0	inside	69	79
BBK26341.1	TMHMM2.0	TMhelix	80	102
BBK26341.1	TMHMM2.0	outside	103	116
BBK26341.1	TMHMM2.0	TMhelix	117	139
BBK26341.1	TMHMM2.0	inside	140	159
BBK26341.1	TMHMM2.0	TMhelix	160	182
BBK26341.1	TMHMM2.0	outside	183	201
BBK26341.1	TMHMM2.0	TMhelix	202	224
BBK26341.1	TMHMM2.0	inside	225	362
BBK26341.1	TMHMM2.0	TMhelix	363	385
BBK26341.1	TMHMM2.0	outside	386	404
BBK26341.1	TMHMM2.0	TMhelix	405	424
BBK26341.1	TMHMM2.0	inside	425	446

ADRB1 beta-1 adrenergic receptor [Gallus gallus] GenBank: BBK26345.2

BBK26345.2 Number of predicted TMHs: 7
BBK26345.2 Exp number of AAs in TMHs: 160.90505
BBK26345.2 Exp number, first 60 AAs: 16.43186
BBK26345.2 Total prob of N-in: 0.00182
BBK26345.2 POSSIBLE N-term signal sequence

BBK26345.2	TMHMM2.0	outside	1	44
BBK26345.2	TMHMM2.0	TMhelix	45	67
BBK26345.2	TMHMM2.0	inside	68	79
BBK26345.2	TMHMM2.0	TMhelix	80	102
BBK26345.2	TMHMM2.0	outside	103	116
BBK26345.2	TMHMM2.0	TMhelix	117	139
BBK26345.2	TMHMM2.0	inside	140	159
BBK26345.2	TMHMM2.0	TMhelix	160	182
BBK26345.2	TMHMM2.0	outside	183	205
BBK26345.2	TMHMM2.0	TMhelix	206	228
BBK26345.2	TMHMM2.0	inside	229	290
BBK26345.2	TMHMM2.0	TMhelix	291	313
BBK26345.2	TMHMM2.0	outside	314	322
BBK26345.2	TMHMM2.0	TMhelix	323	345
BBK26345.2	TMHMM2.0	inside	346	477

ADRB2 beta-2 adrenergic receptor [Gallus gallus] GenBank: BBK26353.1

BBK26353.1 Length: 397
BBK26353.1 Number of predicted TMHs: 7
BBK26353.1 Exp number of AAs in TMHs: 153.64313
BBK26353.1 Exp number, first 60 AAs: 23.00243
BBK26353.1 Total prob of N-in: 0.00236
BBK26353.1 POSSIBLE N-term signal sequence

BBK26353.1	TMHMM2.0	outside	1	28
BBK26353.1	TMHMM2.0	TMhelix	29	51
BBK26353.1	TMHMM2.0	inside	52	63
BBK26353.1	TMHMM2.0	TMhelix	64	86
BBK26353.1	TMHMM2.0	outside	87	105
BBK26353.1	TMHMM2.0	TMhelix	106	128
BBK26353.1	TMHMM2.0	inside	129	148
BBK26353.1	TMHMM2.0	TMhelix	149	171
BBK26353.1	TMHMM2.0	outside	172	194
BBK26353.1	TMHMM2.0	TMhelix	195	217
BBK26353.1	TMHMM2.0	inside	218	268
BBK26353.1	TMHMM2.0	TMhelix	269	291
BBK26353.1	TMHMM2.0	outside	292	300
BBK26353.1	TMHMM2.0	TMhelix	301	320
BBK26353.1	TMHMM2.0	inside	321	397

ADRA2A	alpha-2A adrenergic receptor [Gallus gallus] GenBank: BBK26327.2				
	# BBK26327.2 Length: 444				
	# BBK26327.2 Number of predicted TMHs: 7				
	# BBK26327.2 Exp number of AAs in TMHs: 157.45425				
	# BBK26327.2 Exp number, first 60 AAs: 1.74334				
	# BBK26327.2 Total prob of N-in: 0.00113				
	BBK26327.2	TMHMM2.0	outside	1	59
	BBK26327.2	TMHMM2.0	TMhelix	60	82
	BBK26327.2	TMHMM2.0	inside	83	93
	BBK26327.2	TMHMM2.0	TMhelix	94	116
	BBK26327.2	TMHMM2.0	outside	117	130
	BBK26327.2	TMHMM2.0	TMhelix	131	153
	BBK26327.2	TMHMM2.0	inside	154	173
	BBK26327.2	TMHMM2.0	TMhelix	174	196
	BBK26327.2	TMHMM2.0	outside	197	217
	BBK26327.2	TMHMM2.0	TMhelix	218	240
	BBK26327.2	TMHMM2.0	inside	241	363
	BBK26327.2	TMHMM2.0	TMhelix	364	386
	BBK26327.2	TMHMM2.0	outside	387	400
	BBK26327.2	TMHMM2.0	TMhelix	401	423
	BBK26327.2	TMHMM2.0	inside	424	444

ADRA2B	alpha-2B adrenergic receptor [Gallus gallus] GenBank: BBK26332.1				
	# BBK26332.1 Length: 345				
	# BBK26332.1 Number of predicted TMHs: 7				
	# BBK26332.1 Exp number of AAs in TMHs: 157.66834				
	# BBK26332.1 Exp number, first 60 AAs: 23.0819				
	# BBK26332.1 Total prob of N-in: 0.00002				
	# BBK26332.1 POSSIBLE N-term signal sequence				
	BBK26332.1	TMHMM2.0	outside	1	28
	BBK26332.1	TMHMM2.0	TMhelix	29	51
	BBK26332.1	TMHMM2.0	inside	52	63
	BBK26332.1	TMHMM2.0	TMhelix	64	86
	BBK26332.1	TMHMM2.0	outside	87	100
	BBK26332.1	TMHMM2.0	TMhelix	101	123
	BBK26332.1	TMHMM2.0	inside	124	143
	BBK26332.1	TMHMM2.0	TMhelix	144	164
	BBK26332.1	TMHMM2.0	outside	165	178
	BBK26332.1	TMHMM2.0	TMhelix	179	201
	BBK26332.1	TMHMM2.0	inside	202	265
	BBK26332.1	TMHMM2.0	TMhelix	266	288
	BBK26332.1	TMHMM2.0	outside	289	302
	BBK26332.1	TMHMM2.0	TMhelix	303	325
	BBK26332.1	TMHMM2.0	inside	326	345

ADRB3	beta-3 adrenergic receptor [Gallus gallus] GenBank: BBK26359.1				
	# BBK26359.1 Length: 437				
	# BBK26359.1 Number of predicted TMHs: 7				
	# BBK26359.1 Exp number of AAs in TMHs: 151.94344				
	# BBK26359.1 Exp number, first 60 AAs: 25.32602				
	# BBK26359.1 Total prob of N-in: 0.10698				
	# BBK26359.1 POSSIBLE N-term signal sequence				
	BBK26359.1	TMHMM2.0	outside	1	33
	BBK26359.1	TMHMM2.0	TMhelix	34	56
	BBK26359.1	TMHMM2.0	inside	57	68
	BBK26359.1	TMHMM2.0	TMhelix	69	91
	BBK26359.1	TMHMM2.0	outside	92	105
	BBK26359.1	TMHMM2.0	TMhelix	106	128
	BBK26359.1	TMHMM2.0	inside	129	148
	BBK26359.1	TMHMM2.0	TMhelix	149	168
	BBK26359.1	TMHMM2.0	outside	169	197
	BBK26359.1	TMHMM2.0	TMhelix	198	220
	BBK26359.1	TMHMM2.0	inside	221	271
	BBK26359.1	TMHMM2.0	TMhelix	272	294
	BBK26359.1	TMHMM2.0	outside	295	303
	BBK26359.1	TMHMM2.0	TMhelix	304	326
	BBK26359.1	TMHMM2.0	inside	327	437

Table S6. N_{ST} population analysis data of ADRA1A.

	Site number	106	265	282	417	660	726	735	774	936	1050	1080	1093	1245	1320									
ADRA1A		1	2	3	4	5	6	7	8	9	10	11	12	13	14		ADRA1A							
	A	0	0	0	3	5	0	0	1	0	0	0	6	6	3	9	0.1666667	0.027777778		A	A	21	0.152174	0.0231569
		0	0	0	0.5	0.8333333	0	0	0.1666667	0	0	0	1	1	0.5	0.2857143	0.167				T	43	0.311594	0.0970909
	T	3	4	4	0	0	0	1	0	5	6	0	0	0	0	17	0.3148148	0.099108368		T	G	33	0.23913	0.0571834
		0.5	0.6666667	0.6666667	0	0	0	0.1666667	0	0.8333333	1	0	0	0	0	0.2738095	0.314				C	41	0.297101	0.0882693
Shamo	G	0	0	0	3	1	0	0	5	0	0	0	0	0	3	9	0.1666667	0.027777778		G			1	0.2657005
		0	0	0	0.5	0.1666667	0	0	0.8333333	0	0	0	0	0	0.5	0.1428571	0.167				Total 1	138	H_{Ti}	0.7342995
	C	3	2	2	0	0	6	5	0	1	0	6	0	0	0	19	0.3518519	0.123799726	54	C	Total 2	138		
		0.5	0.3333333	0.3333333	0	0	1	0.8333333	0	0.1666667	0	1	0	0	0	0.297619	0.352							
		1.000	1.000	1.000	1.000	1.000	1.000	1.000	1.000	1.000	1.000	1.000	1.000	1.000	1.000	1.000	1.000	0.278	0.722	*Hsij*				
Hs	Heterozygo	0.500	0.444	0.444	0.500	0.278	0.000	0.278	0.278	0.278	0.000	0.000	0.000	0.000	0.500	0.3333333	0.333	H_{Si}	0.017	N_{STij}				
	A	0	0	0	3	3	0	0	2	0	0	0	3	1	0	12	0.1428571	0.020408163		A				
		0	0	0	0.5	0.5	0	0	0.3333333	0	0	0	0.5	0.1666667	0	0.1481481	0.148							
	T	4	4	4	0	0	1	0	0	6	4	3	0	0	0	26	0.3095238	0.095804989		T				
		0.6666667	0.6666667	0.6666667	0	0	0.1666667	0	0	1	0.6666667	0.5	0	0	0	0.3518519	0.352							
Chicken	G	0	0	0	3	3	0	0	4	0	0	0	3	5	6	24	0.2857143	0.081632653		G				
		0	0	0	0.5	0.5	0	0	0.6666667	0	0	0	0.5	0.8333333	1	0.1851852	0.185							
	C	2	2	2	0	0	5	6	0	0	2	3	0	0	0	22	0.2619048	0.068594104	84	C				
		0.3333333	0.3333333	0.3333333	0	0	0.8333333	1	0	0	0.3333333	0.5	0	0	0	0.3148148	0.315							
		1.000	1.000	1.000	1.000	1.000	1.000	1.000	1.000	1.000	1.000	1.000	1.000	1.000	1.000	1.000	1.000	0.2664399	0.7335601	*Hsij*				
Hs	Heterozygo	0.444	0.444	0.444	0.500	0.500	0.278	0.000	0.444	0.000	0.444	0.500	0.500	0.278	0.000	0.341	0.340	H_{Si}	0.001	N_{STij}				
																		H_{Ti}	0.734					

Table S7. N_{ST} population analysis data of ADRA1B.

	Site number	33	123	387	480	663	773	867	1110	1480										
ADRA1B		1	2	3	4	5	6	7	8	9		ADRA1B								
	A	2	6	0	0	0	0	6	0	6	14	0.3333333	0.111111111		A	A	25	0.297619	0.0885771	
		0.3333333	1	0	0	0	0	1	0	1	0.3333333	0.333				T	12	0.142857	0.0204082	
	T	0	0	6	0	3	0	0	0	0	9	0.2142857	0.045918367		T	G	35	0.416667	0.1736111	
		0	0	1	0	0.5	0	0	0	0	0.2142857	0.2142				C	12	0.142857	0.0204082	
Shamo	G	4	0	0	6	0	6	0	6	0	16	0.3809524	0.145124717		G			1	0.3030045	
		0.6666667	0	0	1	0	1	0	1	0	0.3809524	0.381				Total 1	84	H_{Ti}	0.6969955	
	C	0	0	0	0	3	0	0	0	0	3	0.0714286	0.005102041	42	C	Total 2	84			
		0	0	0	0	0.5	0	0	0	0	0.0714286	0.0714								
		1.000	1.000	1.000	1.000	1.000	1.000	1.000	1.000	1.000	1.000	1.000	0.307	0.693	*Hsij*					
Hs	Heterozygo	0.444	0.000	0.000	0.000	0.500	0.000	0.000	0.000	0.000	0.1349206	0.135	H_{Si}	0.006	N_{STij}					
	A	5	2	0	1	0	1	2	1	5	11	0.2619048	0.068594104		A					
		0.8333333	0.3333333	0	0.1666667	0	0.1666667	0.3333333	0.1666667	0.8333333	0.2619048	0.2619								
	T	0	0	2	0	1	0	0	0	0	3	0.0714286	0.005102041		T					
		0	0	0.3333333	0	0.1666667	0	0	0	0	0.0714286	0.0714								
Chicken	G	1	4	0	5	0	5	4	5	1	19	0.452381	0.204648526		G					
		0.1666667	0.6666667	0	0.8333333	0	0.8333333	0.6666667	0.8333333	0.1666667	0.452381	0.452								
	C	0	0	4	0	5	0	0	0	0	9	0.2142857	0.045918367	42	C					
		0	0	0.6666667	0	0.8333333	0	0	0	0	0.2142857	0.2142								
		1.000	1.000	1.000	1.000	1.000	1.000	1.000	1.000	1.000	1.000	1.000	0.3242630	0.6757370	*Hsij*					
Hs	Heterozygo	0.278	0.444	0.444	0.278	0.278	0.278	0.444	0.278	0.278	0.349	0.349	H_{Si}	0.031	N_{STij}					
													H_{Ti}	0.697						

Table S8. N_{ST} population analysis data of ADRA1D.

Site number		173	216	213	612	1068	1179	1319									
ADRA1D		1	2	3	4	5	6	7		ADRA1D							
	A	0	0	0	0	0	0	3	3	0.0714286	0.005102041		A	A	3	0.035714	0.0012755
		0	0	0	0	0	0	0.5	0.0714286	0.071				T	23	0.27381	0.0749717
	T	5	0	0	0	3	0	0	8	0.1904762	0.036281179		T	G	5	0.059524	0.0035431
		0.8333333	0	0	0	0.5	0	0	0.1904762	0.1905				C	53	0.630952	0.3981009
Shamo	G	1	0	0	0	0	3	0	4	0.0952381	0.009070295		G			1	0.4778912
		0.1666667	0	0	0	0	0.5	0	0.0952381	0.095				Total 1	84	H_{Ti}	0.5221088
	C	0	6	6	6	3	3	3	27	0.6428571	0.413265306	42	C	Total 2	84		
		0	1	1	1	0.5	0.5	0.5	0.6428571	0.643							
		1.000	1.000	1.000	1.000	1.000	1.000	1.000	1.000	1.000	0.464	0.536	*Hsij*				
Hs	Heterozygo	0.278	0.000	0.000	0.000	0.500	0.500	0.500	0.2539683	0.254	H_{Si}	-0.027	N_{STij}				
	A	0	0	0	0	0	0	0	0	0	0		A				
		0	0	0	0	0	0	0	0	0							
	T	6	1	1	2	5	0	0	15	0.3571429	0.12755102		T				
		1	0.1666667	0.1666667	0.3333333	0.8333333	0	0	0.3571429	0.357							
Chicken	G	0	0	0	0	0	1	0	1	0.0238095	0.000566893		G				
		0	0	0	0	0	0.1666667	0	0.0238095	0.024							
	C	0	5	5	4	1	5	6	26	0.6190476	0.383219955	42	C				
		0	0.8333333	0.8333333	0.6666667	0.1666667	0.8333333	1	0.6190476	0.619							
		1.000	1.000	1.000	1.000	1.000	1.000	1.000	1.000	1.000	0.5113379	0.4886621	*Hsij*				
Hs	Heterozygo	0.000	0.278	0.278	0.444	0.278	0.278	0.000	0.222	0.222	H_{Si}	0.064	N_{STij}				

H_{Ti}	0.522

Table S9. N_{ST} population analysis data of ADRA2A.

	Site number	121	162	192	249	255	297	391	579	645	687	747	768	792	816	828	835	843	900	972	1035	1167	1224									
ADRA2A		1	2	3	4	5	6	7	8	9	10	11	12	13	14	15	16	17	18	19	20	21	22		ADRA2A							
	A	5	0	0	2	0	3	0	0	0	2	0	2	0	0	3	1	0	1	3	3	0	0	25	0.1893939	0.0358701		A	A	44	0.166667	0.0277778
		0.8333333	0	0	0.3333333	0	0.5	0	0	0	0.3333333	0	0.3333333	0	0	0.5	0.1666667	0	0.1666667	0.5	0.5	0	0	0.1893939	0.189				T	48	0.181818	0.0330579
	T	0	2	0	0	3	0	3	0	4	0	2	0	0	5	0	0	0	0	0	0	4	0	23	0.1742424	0.0303604		T	G	61	0.231061	0.053389
		0	0.3333333	0	0	0.5	0	0.5	0	0.6666667	0	0.3333333	0	0	0.8333333	0	0	0	0	0	0	0.6666667	0	0.1742424	0.174				C	111	0.420455	0.176782
Shamo	G	1	0	0	0	0	3	0	6	0	0	0	0	0	0	3	5	0	5	3	3	0	0	29	0.219697	0.0482668		G			1	0.2910067
		0.1666667	0	0	0	0	0.5	0	1	0	0	0	0	0	0	0.5	0.8333333	0	0.8333333	0.5	0.5	0	0	0.219697	0.22				Total 1	264	H_{Ti}	0.7089933
	C	0	4	6	4	3	0	3	0	2	4	4	4	6	1	0	0	6	0	0	0	2	6	55	0.4166667	0.1736111	132	C	Total 2	264		
		0	0.6666667	1	0.6666667	0.5	0	0.5	0	0.3333333	0.6666667	0.6666667	0.6666667	1	0.1666667	0	0	1	0	0	0	0.3333333	1	0.4166667	0.417							
		1.000	1.000	1.000	1.000	1.000	1.000	1.000	1.000	1.000	1.000	1.000	1.000	1.000	1.000	1.000	1.000	1.000	1.000	1.000	1.000	1.000	1.000	1.000	1.000	0.288	0.712	*Hsij*				
Hs	Heterozygo	0.278	0.444	0.000	0.444	0.500	0.500	0.500	0.000	0.444	0.444	0.444	0.444	0.000	0.278	0.500	0.278	0.000	0.278	0.500	0.500	0.444	0.000	0.328	0.328	H_{Si}	-0.004	N_{STij}				
	A	2	0	0	1	0	2	0	3	0	1	0	1	0	0	1	0	0	3	1	4	0	0	19	0.1439394	0.0207185		A				
		0.3333333	0	0	0.1666667	0	0.3333333	0	0.5	0	0.1666667	0	0.1666667	0	0	0.1666667	0	0	0.5	0.1666667	0.6666667	0	0	0.1439394	0.1439							
	T	0	0	1	0	2	0	2	0	3	0	3	0	2	4	0	0	2	0	0	0	3	3	25	0.1893939	0.0358701		T				
		0	0	0.1666667	0	0.3333333	0	0.3333333	0	0.5	0	0.5	0	0.3333333	0.6666667	0	0	0.3333333	0	0	0	0.5	0.5	0.1893939	0.1893							
Chicken	G	4	0	0	0	0	4	0	3	0	0	0	0	0	0	5	6	0	3	5	2	0	0	32	0.2424242	0.0587695		G				
		0.6666667	0	0	0	0	0.6666667	0	0.5	0	0	0	0	0	0	0.8333333	1	0	0.5	0.8333333	0.3333333	0	0	0.2424242	0.2424							
	C	0	6	5	5	4	0	4	0	3	5	3	5	4	2	0	0	4	0	0	0	3	3	56	0.4242424	0.1799816	132	C				
		0	1	0.8333333	0.8333333	0.6666667	0	0.6666667	0	0.5	0.8333333	0.5	0.8333333	0.6666667	0.3333333	0	0	0.6666667	0	0	0	0.5	0.5	0.4242424	0.424							
		1.000	1.000	1.000	1.000	1.000	1.000	1.000	1.000	1.000	1.000	1.000	1.000	1.000	1.000	1.000	1.000	1.000	1.000	1.000	1.000	1.000	1.000	1.000	1.000	0.2953398	0.7046602	*Hsij*				
Hs	Heterozygo	0.444	0.000	0.278	0.278	0.444	0.444	0.444	0.500	0.500	0.278	0.500	0.278	0.444	0.444	0.278	0.000	0.444	0.500	0.278	0.444	0.500	0.500	0.374	0.374	H_{Si}	0.006	N_{STij}				

H_{Ti}	0.709

Table S10. N_{ST} population analysis data of ADRA2B.

	Site number	390	411	413	537	629	636	702	717	874	987									
ADRa2B		1	2	3	4	5	6	7	8	9	10		ADRa2B							
	A	2	1	1	4	1	0	0	0	2	0	11	0.1833333	0.033611111		A	A	22	0.183333	0.0336111
		0.3333333	0.1666667	0.1666667	0.6666667	0.1666667	0	0	0	0.3333333	0	0.1833333	0.183				T	6	0.05	0.0025
	T	0	0	0	0	0	0	0	0	0	0	0	0	0		T	G	74	0.616667	0.3802778
		0	0	0	0	0	0	0	0	0	0	0	0				C	18	0.15	0.0225
Shamo	G	4	5	5	2	5	0	6	6	4	0	37	0.6166667	0.380277778		G			1	0.4388889
		0.6666667	0.8333333	0.8333333	0.3333333	0.8333333	0	1	1	0.6666667	0	0.6166667	0.6166				Total_1	120	H_{Ti}	0.5611111
	C	0	0	0	0	0	6	0	0	0	6	12	0.2	0.04	60	C	Total_2	120		
		0	0	0	0	0	1	0	0	0	1	0.2	0.2							
		1.000	1.000	1.000	1.000	1.000	1.000	1.000	1.000	1.000	1.000	1.000	1.000	0.454	0.546	*Hsij*				
Hs	Heterozygo	0.4444444	0.2777778	0.2777778	0.4444444	0.2777778	0	0	0	0.4444444	0	0.2407407	0.241	H_{Si}	0.027	N_{STij}				
	A	1	0	0	5	0	0	2	3	0	0	11	0.1833333	0.033611111		A				
		0.1666667	0	0	0.8333333	0	0	0.3333333	0.5	0	0	0.2037037	0.204							
	T	0	0	0	0	0	2	0	0	0	4	6	0.1	0.01		T				
		0	0	0	0	0	0.3333333	0	0	0	0.6666667	0.037037	0.037							
Chicken	G	5	6	6	1	6	0	4	3	6	0	37	0.6166667	0.380277778		G				
		0.8333333	1	1	0.1666667	1	0	0.6666667	0.5	1	0	0.6851852	0.685							
	C	0	0	0	0	0	4	0	0	0	2	6	0.1	0.01	60	C				
		0	0	0	0	0	0.6666667	0	0	0	0.3333333	0.0740741	0.074							
		1.000	1.000	1.000	1.000	1.000	1.000	1.000	1.000	1.000	1.000	1.000	1.000	0.4338889	0.5661111	*Hsij*				
Hs	Heterozygo	0.278	0.000	0.000	0.278	0.000	0.444	0.444	0.500	0.000	0.444	0.216	0.216	H_{Si}	-0.009	N_{STij}				
														H_{Ti}	0.561					

Table S11. N_{ST} population analysis data of ADRB1.

	Site number	492	874	1200	1208	1328	1330	1334	1396									
ADRB1		1	2	3	4	5	6	7	8		ADRB1							
	A	0	0	0	6	2	0	4	0	12	0.25	0.0625		A	A	23	0.239583	0.0574002
		0	0	0	1	0.3333333	0	0.6666667	0	0.25	0				T	17	0.177083	0.0313585
	T	0	6	0	0	0	0	0	4	10	0.20833333	0.043402778		T	G	37	0.385417	0.148546
		0	1	0	0	0	0	0	0.6666667	0.20833333	0				C	19	0.197917	0.039171
Shamo	G	0	0	6	0	4	6	2	0	18	0.375	0.140625		G			1	0.2764757
		0	0	1	0	0.6666667	1	0.3333333	0	0.375	0				Total	96	H_{Ti}	0.7235243
	C	6	0	0	0	0	0	0	2	8	0.16666667	0.027777778	48	C	Total	96		
		1	0	0	0	0	0	0	0.3333333	0.16666667	0							
		1.000	1.000	1.000	1.000	1.000	1.000	1.000	1.000	1.000	1.000	0.274	0.726	*Hsij*				
Hs	Heterozygo	0	0	0	0	0.444	0.000	0.444	0.444	0.16666667	0.222	H_{Si}	-0.003	NSTij				
	A	0	0	2	2	6	1	0	0	11	0.22916667	0.052517361		A				
		0	0	0.3333333	0.3333333	1	0.1666667	0	0	0.22916667	0							
	T	2	5	0	0	0	0	0	0	7	0.14583333	0.021267361		T				
		0.3333333	0.8333333	0	0	0	0	0	0	0.14583333	0							
Chicken	G	0	0	4	4	0	5	6	0	19	0.39583333	0.156684028		G				
		0	0	0.6666667	0.6666667	0	0.8333333	1	0	0.39583333	0							
	C	4	1	0	0	0	0	0	6	11	0.22916667	0.052517361	48	C				
		0.6666667	0.1666667	0	0	0	0	0	1	0.22916667	0							
		1.000	1.000	1.000	1.000	1.000	1.000	1.000	1.000	1.000	1.000	0.2829861	0.7170139	*Hsij*				
Hs	Heterozygo	0.444	0.278	0.444	0.444	0.000	0.278	0.000	0.000	0.23611111	0.239	H_{Si}	0.009	N_{STij}				
												H_{Ti}	0.724					

Table S12. N_{ST} population analysis data of ADRB2.

	Site number	43	131	132	612	624	648	695	830	882									
ADRB2		1	2	3	4	5	6	7	8	9		ADRB2							
	A	2	0	0	0	0	0	4	0	0	6	0.1111111	0.012345679		A	A	12	0.111111	0.0123457
		0.3333333	0	0	0	0	0	0.6666667	0	0	0.1111111	0.11				T	29	0.268519	0.0721022
	T	0	2	4	2	4	2	0	1	0	15	0.2777778	0.077160494		T	G	12	0.111111	0.0123457
		0	0.3333333	0.6666667	0.3333333	0.6666667	0.3333333	0	0.1666667	0	0.2777778	0.28				C	55	0.509259	0.259345
Shamo	G	4	0	0	0	0	0	2	0	0	6	0.1111111	0.012345679		G			1	0.3561385
		0.6666667	0	0	0	0	0	0.3333333	0	0	0.1111111	0.11				Total	108	H$_{Ti}$	0.6438615
	C	0	4	2	4	2	4	0	5	6	27	0.5	0.25	54	C	Total	108		
		0	0.6666667	0.3333333	0.6666667	0.3333333	0.6666667	0	0.8333333	1	0.5	0.5							
		1.000	1.000	1.000	1.000	1.000	1.000	1.000	1.000	1.000	1.000	1.000	0.352	0.648	*Hsij*				
Hs	Heterozygo	0.4444444	0.4444444	0.4444444	0.4444444	0.4444444	0.4444444	0.4444444	0.2777778	0	0.3765432	0.377	H_{Si}	-0.007	N$_{STij}$				
	A	0	0	0	0	0	0	6	0	0	6	0.1111111	0.012345679		A				
		0	0	0	0	0	0	1	0	0	0.1111111	0.11							
	T	0	0	6	0	6	0	0	0	2	14	0.2592593	0.067215364		T				
		0	0	1	0	1	0	0	0	0.3333333	0.2592593	0.26							
Chicken	G	6	0	0	0	0	0	0	0	0	6	0.1111111	0.012345679		G				
		1	0	0	0	0	0	0	0	0	0.1111111	0.11							
	C	0	6	0	6	0	6	0	6	4	28	0.5185185	0.268861454	54	C				
		0	1	0	1	0	1	0	1	0.6666667	0.5185185	0.52							
		1.000	1.000	1.000	1.000	1.000	1.000	1.000	1.000	1.000	1.000	1.000	0.3607682	0.6392318	*Hsij*				
Hs	Heterozygo	0.000	0.000	0.000	0.000	0.000	0.000	0.000	0.000	0.444	0.049	0.049	H_{Si}	0.007	N$_{STij}$				
													H_{Ti}	0.644					
